# HERA-GITRL activates T cells and promotes anti-tumor efficacy independent of FcγR-binding functionality

**DOI:** 10.1186/s40425-019-0671-4

**Published:** 2019-07-19

**Authors:** David M. Richards, Viola Marschall, Katharina Billian-Frey, Karl Heinonen, Christian Merz, Mauricio Redondo Müller, Julian P. Sefrin, Matthias Schröder, Jaromir Sykora, Harald Fricke, Oliver Hill, Christian Gieffers, Meinolf Thiemann

**Affiliations:** 1 Present address: Biotest AG, Dreieich, Germany; 2 Present address: SOTIO, Prague, Czech Republic; 3 Research and Development, Apogenix AG, Im Neuenheimer Feld 584, 69120 Heidelberg, Germany

**Keywords:** Single-chain GITRL, scGITRL-RBD, GITR, Agonist, TNFSF, CD357, HERA

## Abstract

**Background:**

Glucocorticoid-induced TNFR-related protein (TNFRSF18, GITR, CD357), expressed by T cells, and its ligand (TNFSF18, GITRL), expressed by myeloid populations, provide co-stimulatory signals that boost T cell activity. Due to the important role that GITR plays in regulating immune functions, agonistic stimulation of GITR is a promising therapeutic concept. Multiple strategies to induce GITR signaling have been investigated. The limited clinical efficacy of antibody-based GITR agonists results from structural and functional characteristics of antibodies that are unsuitable for stimulating the well-defined trimeric members of the TNFRSF.

**Methods:**

To overcome limitations of antibody-based TNFRSF agonists, we have developed HERA-GITRL, a fully human *hex*avalent TNF *r*eceptor *a*gonist (HERA) targeting GITR and mimicking the natural signaling concept. HERA-GITRL is composed of a trivalent but single-chain GITRL-receptor-binding-domain (scGITRL-RBD) unit fused to an IgG1 derived silenced Fc-domain serving as dimerization scaffold. A specific mouse surrogate, mmHERA-GITRL, was also generated to examine in vivo activity in respective mouse tumor models.

**Results:**

For functional characterization of HERA-GITRL in vitro, human immune cells were isolated from healthy-donor blood and stimulated with anti-CD3 antibody in the presence of HERA-GITRL. Consistently, HERA-GITRL increased the activity of T cells, including proliferation and differentiation, even in the presence of regulatory T cells.

In line with these findings, mmHERA-GITRL enhanced antigen-specific clonal expansion of both CD4+ (OT-II) and CD8+ (OT-I) T cells in vivo while having no effect on non-specific T cells. In addition, mmHERA-GITRL showed single-agent anti-tumor activity in two subcutaneous syngeneic colon cancer models (CT26wt and MC38-CEA). Importantly, this activity is independent of its FcγR-binding functionality, as both mmHERA-GITRL with a functional Fc- and a silenced Fc-domain showed similar tumor growth inhibition.

Finally, in a direct in vitro comparison to a bivalent clinical benchmark anti-GITR antibody and a trivalent GITRL, only the hexavalent HERA-GITRL showed full biological activity independent of additional crosslinking.

**Conclusion:**

In this manuscript, we describe the development of HERA-GITRL, a true GITR agonist with a clearly defined mechanism of action. By clustering six receptor chains in a spatially well-defined manner, HERA-GITRL induces potent agonistic activity without being dependent on additional FcγR-mediated crosslinking.

**Electronic supplementary material:**

The online version of this article (10.1186/s40425-019-0671-4) contains supplementary material, which is available to authorized users.

## Background

Strategies to boost anti-tumor immune responses are among the most promising new developments in oncology. This is due to important characteristics of the adaptive immune system; including, antigen-specificity, adaptability and ability to generate long-lasting memory [1]. These functions are orchestrated by a complex and delicately balanced system of stimulatory and inhibitory signals. The tumor necrosis factor superfamily (TNFSF) and their cognate receptors, collectively called the TNF receptor superfamily (TNFRSF) play key roles in maintaining this balance [2–4]. The TNFSF consists of 19 ligands, each binding to one or more of the 29 members of the TNFRSF [4, 5]. These TNFSF/TNFRSF proteins are expressed by a wide variety of immune cells including T cells and antigen-presenting cell (APC) populations, as well as by tumor cells themselves [2, 4]. This diverse expression pattern highlights the critical role that they play in the anti-tumor immune response.

Glucocorticoid-induced TNFR-related protein (TNFRSF18, GITR, CD357), is expressed at low levels on resting T cells, natural killer (NK) cells and some myeloid cell populations. It is expressed at high levels by activated T cells and regulatory T (Treg) cells [6, 7]. GITR signaling, induced by its ligand (TNFSF18, GITRL), expressed by APC populations, plays an important role in regulating immune responses by providing co-stimulatory signals to boost T cell activation, differentiation, survival and memory formation [2, 8]. GITR is therefore an especially important target for immunotherapy.

TNFRSF signaling is a structurally well-defined event that requires very specific receptor clustering and trimerization [4, 9–11]. While the ligands naturally exist as trivalent functional units, the receptors are separated on the cell surface and need to be organized into functional trimeric assemblies. This precise organization is mediated by the trivalent ligand units upon receptor binding, and necessary for effective signaling [12].

TNFRSF members have been key immunotherapeutic targets for over 20 years. Multiple strategies to induce GITR signaling are currently being investigated and they can be broadly grouped into GITRL-based (natural ligand mimetics) or agonistic antibody-based approaches [3, 9]. The limited clinical efficacy of agonistic anti-GITR antibodies results from structural and functional characteristics of antibodies, including the presence of only two target-binding sites per molecule, that are unsuitable for stimulating the TNFRSF [9–11]. We have previously described the construction and biological activity of hexavalent TNF receptor agonists (HERA) targeting TRAIL receptors [13], CD27 [14] and CD40 [15]. The unique molecular layout and binding mode of HERA-TRAIL/APG350, HERA-CD27L and HERA-CD40L conferred potent biological activity that was superior to conventional agonistic antibodies and independent of additional crosslinking via Fcγ receptors (FcγR) [9, 13–15].

Here, we describe the development of a novel agonistic HERA molecule targeting GITR. The underlying HERA-GITRL structure overcomes significant limitations of bivalent antibody-based approaches because it mimics the natural trimeric ligand, thus inducing optimal trimeric assembly of the GITR receptors.

## Methods

Detailed methods and statistical tests can be found in Additional file 1: Supplementary Methods.

## Results

### Structure, design and production of a novel hexavalent GITR agonist (HERA-GITRL)

**Fig. 1:**
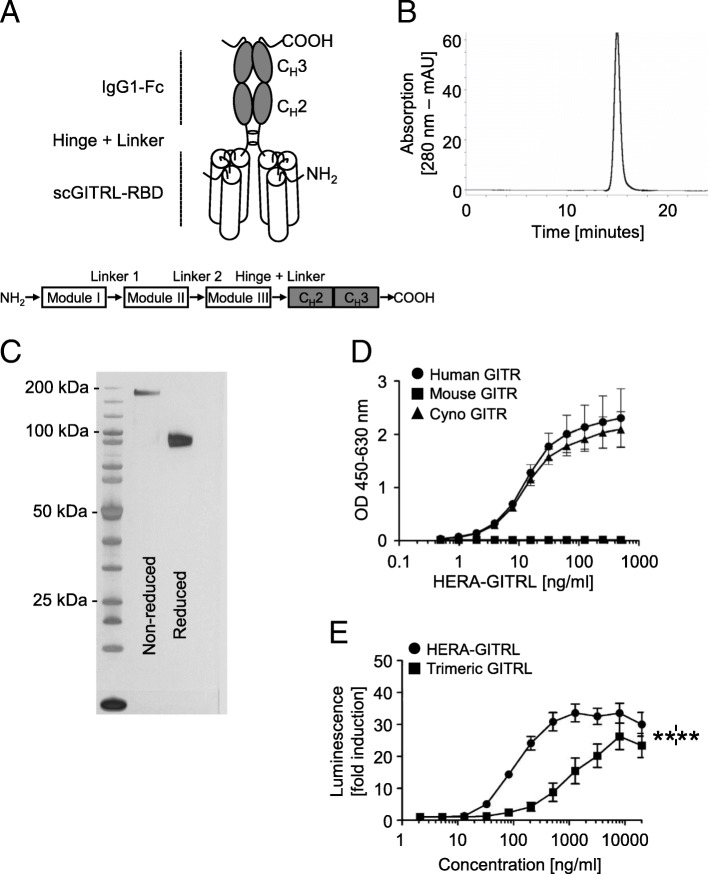
Structure, production and activity of HERA-GITRL. **a** Schematic depiction of the structure of the hexavalent HERA-GITRL. Three copies of a GITRL protomer sub-sequence (called the GITRL-receptor binding domain or GITRL-RBD) were combined into one single chain (sc) polypeptide. This generated a trivalent scGITRL-RBD, which was fused to the Fc part of a human IgG1-mutein to create a hexavalent scGITRL-RBD dimer. **b, c** Purification was accomplished by a two-step process combining AFC followed by preparative SEC. **b** For analytical SEC; purified HERA-GITRL was detected by online measurement of absorption at 280 nm. Content of monomer and aggregates was calculated as the AUC from the elution profile of the SEC. HERA-GITRL eluted as a single peak and showed no detectable aggregates. **c** Purity and aggregation status of purified hexavalent HERA-GITRL was also assessed by non-reducing and reducing SDS-PAGE. **d** ELISA showing binding of hexavalent HERA-GITRL to immobilized human, mouse and cynomolgus monkey GITR-Fc. Plate-bound GITR-Fc was probed with the indicated formats and concentrations. Receptor-bound HERA-GITRL was detected via a Strep-Tag II-specific antibody conjugated to horseradish peroxidase. Values are mean OD (*n* = 3) ± S.D. at a wavelength of 450 nm (with a 630 nm correction). Representative data from three independent experiments are shown. **e** NFκB-luc2/GITR-expressing Jurkat cells were incubated with the indicated concentrations of HERA-GITRL or trimeric GITRL at 37 °C. After six hours, luminescence was measured and the fold induction in luciferase activity was calculated in order to compare multiple experiments. Data are shown as mean values (± S.D.) from three independent experiments. A two-way ANOVA plus post hoc Bonferroni multiple comparisons analysis was conducted to compare the effects of treatment and concentration on luminescence. ****p < 0.0001

To engineer the hexavalent human GITR agonist, HERA-GITRL, we designed a single-polypeptide chain with three copies of a GITRL (TNFSF18) protomer sub-sequence (the construction principles are outlined in Fig. 1a). Specifically, three copies from the human GITRL comprising amino acids E56-S177 are interconnected with two glycine-serine based linkers 8 amino acids in length. The resulting trivalent single chain-GITRL-receptor-binding-domain (scGITRL-RBD) is fused to the Fc-part of a human IgG1-mutein, which is deficient for FcγR binding. For the mouse surrogate GITRL constructs (mmHERA-GITRL), S43-S177 of the mouse GITRL and the mouse IgG2a-Fc domain were used. The mouse surrogates were engineered in two variants with either a FcγR-binding deficient or FcγR-binding competent (labeled “Fc+”) IgG2a-Fc domain. All preclinical HERA ligands included a C-terminal Strep-Tag II for purification of the proteins. Secretory pathway-based expression was achieved by adding an appropriate signal-peptide to the N-terminus.

All HERA-GITRL and mmHERA-GITRL constructs were expressed in Chinese Hamster Ovary cells (CHO-S) and purified by a two-step process combining Streptactin affinity purification (AFC) followed by preparative size-exclusion chromatography (SEC) both performed under physiological buffer conditions at pH 7.4. Purity and integrity of the proteins were confirmed by analytical SEC as well as denaturing and non-denaturing SDS-PAGE (Fig. 1b, c and Additional file 2: Figure S1A, B). As shown, the lab-scale production process yielded defined and stable products devoid of contaminating proteins and product related aggregates. Furthermore, the stability of HERA-GITRL, measured by receptor binding (ELISA), SDS-PAGE, analytical SEC and a thermal shift stability assay, was tested under a variety of different storage and pH conditions in addition to heat stress and freeze/thaw stability. It was found to be very stable and suitable for standard large-scale production processes (Additional file 2: Table S1).

### Target binding properties and cellular activity of HERA-GITRL

In order to demonstrate functional assembly, purified HERA-GITRL was tested for binding to recombinant human, mouse and cynomolgus monkey GITR-Fc by ELISA (Fig. 1d). HERA-GITRL was able to bind to immobilized human and cynomolgus monkey, but not mouse, GITR-Fc demonstrating functional assembly of the respective receptor binding domains and confirming that cynomolgus monkey is a relevant species for further studies. Binding to and dissociation from these receptors were measured in real time, and the respective equilibrium binding constants (K_D_) were calculated (Additional file 2: Table S2). These species-specific results are consistent with previously published data and confirmed that a specific mouse surrogate mmHERA-GITRL needed to be developed [5]. Binding of mmHERA-GITRL to a panel of GITR-Fc proteins demonstrated functional assembly of mmHERA-GITRL and species-specific binding (Additional file 2: Figure S1C and Fig. S2A).

Next, functional binding of HERA-GITRL was evaluated using a reporter cell assay. NFκB-luc2/GITR-expressing Jurkat cells were incubated with various concentrations of HERA-GITRL or a recombinant trimeric human GITRL and after six hours of culture, luminescence was measured. HERA-GITRL showed a strong agonistic activity that was significantly higher than trimeric GITRL over a broad range of concentrations (Fig. 1e).

### HERA-GITRL enhances human T cell activation following stimulation in vitro

**Fig. 2:**
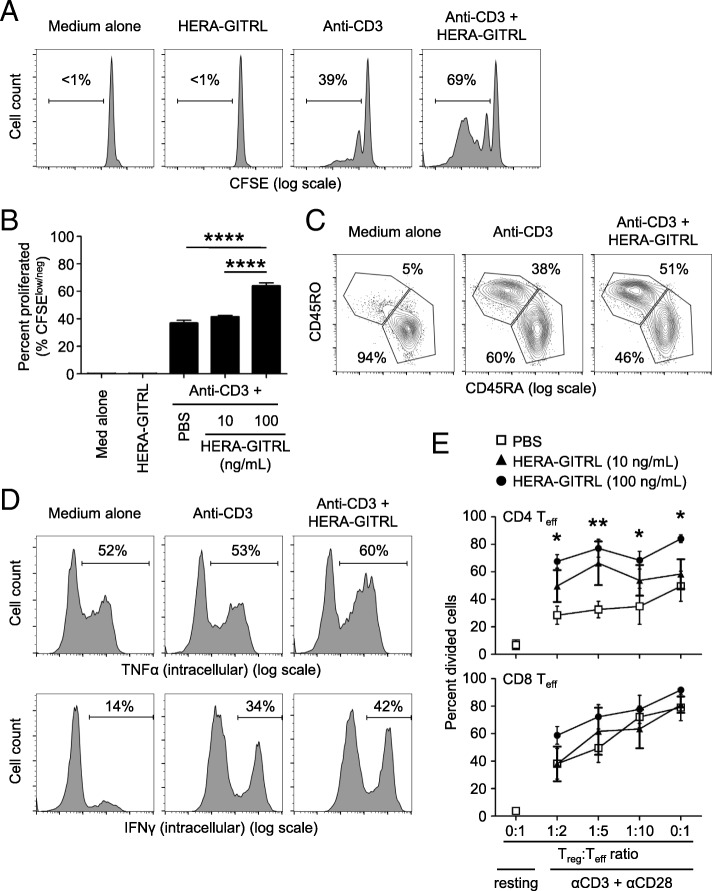
HERA-GITRL enhances human T cell activation following stimulation in vitro. **a, b** Naïve CD4+ T cells were isolated from the peripheral blood of healthy volunteers, labeled with CFSE and stimulated with anti-CD3 antibody (or medium) in the presence of HERA-GITRL or vehicle control (PBS), as indicated. On day five, T cells were harvested and examined by FCM. Representative histograms (**a**) (HERA-GITRL at 100 ng/mL) and quantified data (**b**) (mean ± S.D.) are shown. Although not labeled, all anti-CD3 treated groups were significantly different from the medium alone and HERA-GITRL alone groups. **c** Naïve CD4+ T cells were stimulated with anti-CD3 antibody (or medium) in the presence of HERA-GITRL (100 ng/mL) or vehicle control (PBS), as indicated. On day five, T cells were harvested, stained for surface expression of CD45RO and CD45RA and examined by FCM. **d** Naïve CD4+ T cells were stimulated with anti-CD3 antibody (or medium) in the presence of HERA-GITRL (100 ng/mL) or vehicle control (PBS), as indicated. On day six, T cells were stained for intracellular expression of IFNγ and TNFα and examined by FCM. **e** Treg cells and Teff cells isolated from human PBMCs were cultured together at different ratios (Treg:Teff) and stimulated with anti-CD3 and anti-CD28 antibodies in the presence of IL-2 and ATRA or left unstimulated (resting). HERA-GITRL (10 or 100 ng/mL) or vehicle control (PBS) was added immediately. On day five, T cells were harvested and CTV expression by Teff cells was examined by FCM. Numbers indicate the percentage of cells within the defined region. Representative plots, gated on live single cells, from the median sample of triplicates from at least three independent experiments are shown (**a**, **c**, **d**). The *p* values represent comparisons between samples using a one-way (**b**) or two-way (**e**) ANOVA plus post hoc Bonferroni multiple comparisons test. *p < 0.05, **p < 0.01, ****p < 0.0001

To examine the biological activity of HERA-GITRL, we isolated naïve CD4+ T cells from the peripheral blood of healthy volunteers and stimulated them in the presence of various concentrations of HERA-GITRL. Naïve T cells require a specific T cell receptor (TCR)-mediated signal as well as a second set of “co-stimulatory” signals in order to achieve full activation and differentiation. Here, naïve CD4+ T cells were stimulated with plate-bound anti-CD3 antibody (1 μg/mL) (“signal one”) in the presence of HERA-GITRL (10 or 100 ng/mL). Addition of HERA-GITRL significantly increased the proliferative response of CD4+ T cells stimulated by anti-CD3 antibody (Fig. 2a, b). Corresponding changes in the expression of the classic differentiation markers, CD45RA and CD45RO, were also observed following treatment with HERA-GITRL in combination with anti-CD3 antibody (Fig. 2c). In addition, CD4+ T cells activated in the presence of HERA-GITRL also increased intracellular accumulation of the inflammatory cytokines IFNγ and TNFα (Fig. 2d).

Because regulatory T (Treg) cells play an important role in suppressing immune responses and express high levels of GITR, we decided to investigate the effects of HERA-GITRL treatment on Treg cell activity. Treatment of Treg cells isolated from peripheral blood with various doses of HERA-GITRL did not influence Treg cell survival or proliferation (data not shown). However, an indirect effect on Treg cell function was observed following HERA-GITRL treatment of standard Treg cell suppression assays. (Fig. 2e). Suppression of effector CD4+ T cell proliferation was significantly inhibited by HERA-GITRL treatment. Effector CD8+ T cells also showed an increase in proliferation in the presence of HERA-GITRL. Importantly, treatment with HERA-GITRL alone did not produce any response in unstimulated T cells, suggesting that an antigen-specific and TCR-mediated signal is required for the GITR-mediated enhancement of T cell activity (Fig. 2a, b and data not shown).

### Pharmacokinetics of HERA-GITRL in mouse and cynomolgus monkey

We assessed in vivo stability and pharmacokinetic (PK) parameters of HERA-GITRL. Female CD-1 mice were treated with 1 or 10 mg/kg body weight (b.w.) HERA-GITRL as a single intravenous (i.v.) bolus injection (Additional file 2: Figure S3A). The terminal serum half-life of HERA-GITRL in mice was calculated to be 86 to 97 h (Additional file 2: Table S3). Since human HERA-GITRL binds to cynomolgus monkey GITR, we also performed a PK study in this relevant species (Additional file 2: Figure S3B). The terminal serum half-life of HERA-GITRL in cynomolgus monkeys was calculated to be 37 h after a single i.v. injection of 1 mg/kg b.w. (Additional file 2: Table S3). In addition, no toxicological signs related to the administration of HERA-GITRL were observed during the in-life phase of this PK study (data not shown). PK parameters of the mouse surrogates, mmHERA-GITRL and mmHERA-GITRL (Fc+), were also examined in CD-1 mice (Additional file 2: Figure S2B, C). For mmHERA-GITRL, the terminal serum half-life was calculated to be 7.4 h after a single i.v. injection of 1 mg/kg b.w. (Additional file 2: Table S4). The FcγR-binding competent mmHERA-GITRL (Fc+) had a half-life of 18.3 h.

### mmHERA-GITRL significantly boosts the antigen-specific CD4+ and CD8+ T cell response in vivo

**Fig. 3:**
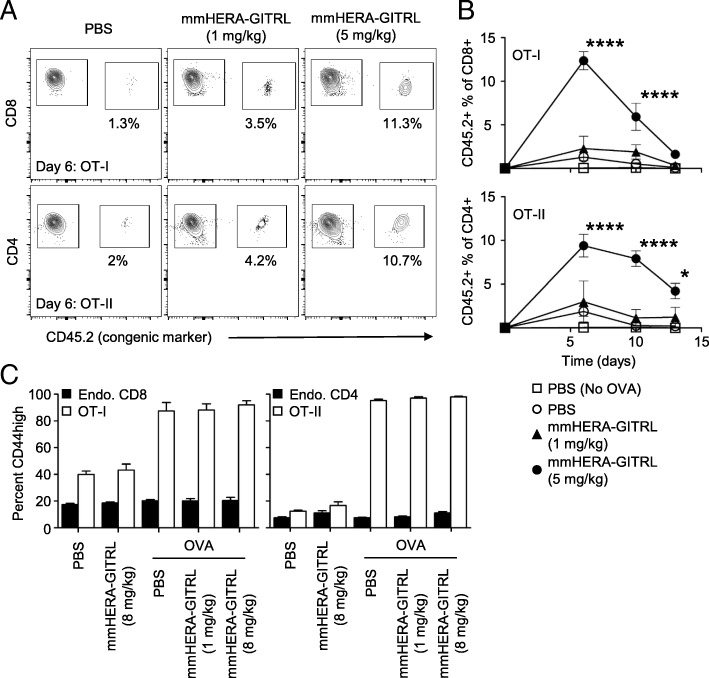
mmHERA-GITRL significantly boosts the antigen-specific T cell response in vivo without affecting endogenous T cells. **a, b** C57Bl/6 mice were adoptively transferred with 2 × 10^6^ (each) CD8+ OT-I T cells and CD4+ OT-II T cells, challenged with OVA protein (or PBS) and treated with a single injection of mmHERA-GITRL (1 or 5 mg/kg b.w.) or vehicle control (PBS). Serial blood samples were obtained from each animal over a two-week period and OT-I/OT-II T cells were quantified by FCM using CD8/CD4 and a congenic marker (CD45.2). A representative time point is shown for day 6 **(a)** and the entire time course is quantified (**b**). **a** Gated regions indicate transferred T cells (right polygon) and endogenous/recipient T cells (left polygon). Numbers indicate the percentage of cells within the OT-I/OT-II T cell region. Representative contour plots, gated on live single CD8+ or CD4+ T cells, from the median sample of triplicates from at least three independent experiments are shown. **b** Day 0 value represents historical adoptive transfer data. Each symbol represents the mean (*n* = 3) ± S.D. A two-way ANOVA plus post hoc Bonferroni multiple comparisons analysis was conducted to compare the effects of treatment and time on antigen-specific T cell clonal expansion. The *p* values represent comparisons between 5 mg/kg mmHERA-GITRL and PBS. *p < 0.05, ****p < 0.0001. **c** In a separate experiment, which included mmHERA-GITRL (8 mg/kg b.w.) treatment alone, blood samples were obtained from each animal on day six, cells were stained for surface expression of CD44 and the percentage CD44high (mean ± S.D.) is shown. One-way ANOVA plus post hoc Bonferroni multiple comparisons analysis was conducted on all treatment groups. No significant changes in CD44 expression were seen in the endogenous T cell populations (“Endo.”). Although not labeled, CD44 expression on OT-I/OT-II T cells in all groups treated with OVA are significantly different from non-OVA treated groups

As HERA-GITRL binds to human and cynomolgus monkey GITR, but not to mouse GITR, a mouse surrogate mmHERA-GITRL was developed and used for in vivo studies. We first assessed the activity of mmHERA-GITRL in vivo using the classic antigen-specific adoptive transfer system [16]. This system allows for the investigation of a more realistically sized antigen-specific T cell population (around 1%) while simultaneously having a large population of non-specific T cells to examine for non-antigen-specific effects. Since many T cells express GITR, there is potential for non-specific and bystander T cell activation. These potential side effects were examined by comparing chicken ovalbumin (OVA)-specific and non-specific CD4+ and CD8+ T cells in the same environment. Since the OVA-specific T cell frequency in wildtype C57Bl/6 mice is very low (on average, less than 1000 cells per mouse [17]), we adoptively transferred 2 × 10^6^ OVA-specific CD8+ “OT-I” and CD4+ “OT-II” T cells to increase the resolution. Naïve Ly5.1 (CD45.1+) recipient mice were adoptively transferred and challenged with OVA protein plus a single injection of mmHERA-GITRL (1 or 5 mg/kg b.w. and 1 or 8 mg/kg b.w.). Serial blood samples were obtained and representative examples of OT-I and OT-II T cell clonal expansion are shown (Fig. 3a). As shown in Fig. 3b, both CD8+ and CD4+ antigen-specific T cells underwent significant clonal expansion during the first week following antigen challenge and treatment with the higher dose of mmHERA-GITRL. Treatment with PBS or 1 mg/kg b.w. mmHERA-GITRL resulted in only a minimal T cell response compared to transfer alone.

In order to examine the specificity of mmHERA-GITRL, expression of CD44, a differentiation/maturation marker, was measured following treatment with HERA-GITRL (Fig. 3c). In the presence of OVA, the antigen-specific CD8+ and CD4+ T cells upregulated surface expression of CD44. In contrast, the endogenous CD8+ and CD4+ T cells did not respond in any of the conditions tested, even when 10 to 15% of the neighboring T cells were vigorously responding to OVA plus mmHERA-GITRL. Importantly, the antigen-specific T cells did not respond to treatment with HERA-GITRL in the absence of their cognate antigen (i.e., OVA) (Fig. 3c).

### mmHERA-GITRL demonstrates significant in vivo efficacy in syngeneic mouse tumor models independent of FcγR-binding functionality

T cell activation assays are well suited for proof of concept and mechanism of actions studies, however, it is important for anti-cancer compounds to show in vivo efficacy in syngeneic mouse tumor models. The standard colon carcinoma model CT26wt, known to be highly immunogenic and generally responsive to immunotherapy, was used to investigate the potency of mmHERA-GITRL treatment.

This model was also used to compare true agonistic activity to the mixed mode of action, agonistic plus FcγR-mediated activity, seen with therapeutic antibodies. The Fc domain of therapeutic antibodies is responsible for mediating a range of antibody effector functions such as antibody-dependent cell-mediated cytotoxicity (ADCC) and phagocytosis (ADCP). In fact, the anti-tumor activity of anti-GITR antibodies of the IgG sub-class has been reported to be dependent on such functions mediated by FcγR [18]. In contrast, HERA-GITRL has been designed to be effective in the absence of FcγR-binding functionality. In order to study the dependence of anti-tumor activity on FcγR binding, we compared the standard mmHERA-GITRL to mmHERA-GITRL (Fc+), which contains a functional Fcγ-receptor-binding domain.

**Fig. 4:**
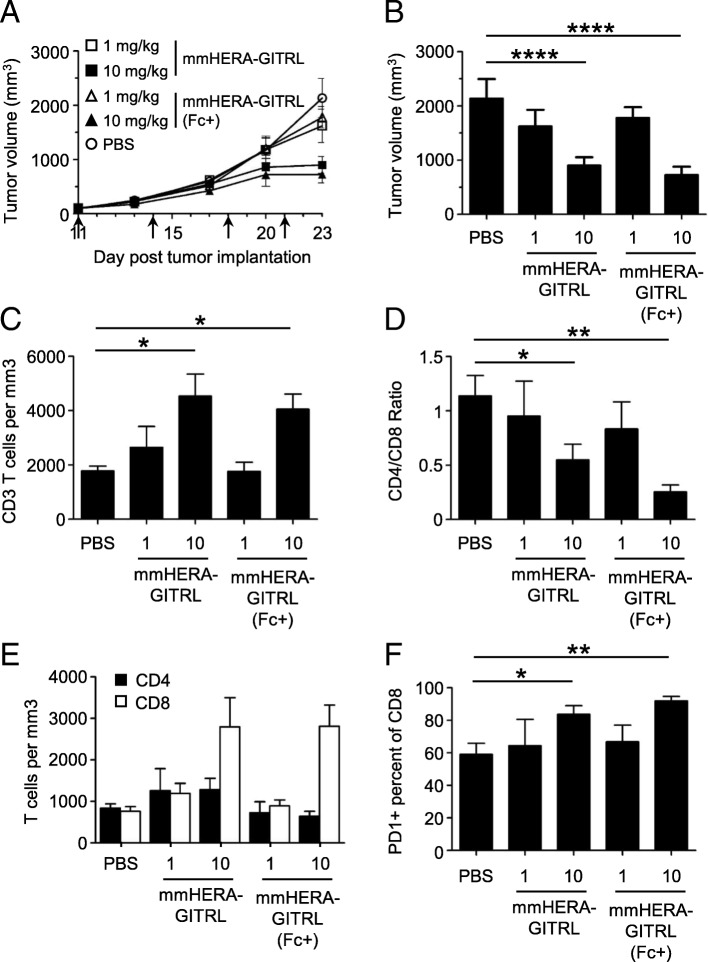
mmHERA-GITRL demonstrates single-agent in vivo efficacy that is independent of FcγR binding. **a - f** Freshly cultured CT26wt tumor cells (5 × 10^5^ in 100 μl RPMI) were implanted s.c. into the right flank of 6-week-old female BALB/c mice. Tumor volume was determined twice weekly by caliper measurement. Mice were randomized on day 11 into groups of 6 mice per treatment group with a mean primary tumor volume of 101 mm^*3*^. All animals were treated (i.v.) with mmHERA-GITRL, mmHERA-GITRL (Fc+) (both at 1 or 10 mg/kg b.w.) or vehicle control (PBS) on days 11, 14, 18 and 21, indicated by the arrows. The in-life phase of the study finished on day 23 following tumor implantation. **a, b** Each symbol or column represents the mean (*n* = 6) ± S.D. **b** Shows the tumor volume on day 23. A two-way ANOVA plus post hoc Bonferroni multiple comparisons analysis was conducted to compare the effects of treatment and time on tumor growth. ****p < 0.0001. **c – f** On day 23, tumors were harvested, stained for surface marker expression and examined by FCM. **c** The total number of CD3+ T cells per mm^*3*^ of tumor tissue is shown. **d** The ratio of CD4+ to CD8+ T cells is shown. **e** The total number of CD4+ (closed boxes) and CD8+ (open boxes) T cells per mm^*3*^ of tumor tissue is shown. **f** The percentage of PD1+ cells is shown for the CD8+ T cell population. The *p* values represent comparisons between samples using a one-way ANOVA plus post hoc Bonferroni multiple comparisons test. *p < 0.05, **p < 0.01. Although not labeled, there are no significant differences between mmHERA-GITRL and mmHERA-GITRL (Fc+) at either dose tested

CT26wt tumor cells were implanted (subcutaneous, s.c.) and mice were randomized into groups 11 days later with a mean primary tumor volume of approximately 100 mm^3^. All mice were treated (i.v.) with mmHERA-GITRL, mmHERA-GITRL (Fc+) (both at 1 or 10 mg/kg b.w.) or vehicle control (PBS) on days 11, 14, 18 and 21. Treatment with both mmHERA-GITRL and mmHERA-GITRL (Fc+) resulted in similar dose-dependent tumor-growth inhibition (TGI) compared to the control group, 58% TGI and 66% TGI, respectively, at 10 mg/kg b.w. (Fig. 4a, b and Additional file 2: Figure S4A).

In addition to TGI, significant changes were also observed in the tumor-infiltrating T cell compartment following treatment with mmHERA-GITRL and mmHERA-GITRL (Fc+). There was a significant and similar increase in the number of tumor-infiltrating T cells following treatment at 10 mg/kg b.w. for both compounds (Fig. 4c). This increase was predominantly mediated by an increase in CD8+ T cell enrichment, which is highlighted by the significant change in the CD4/CD8 ratio as well as a three-fold increase in CD8+ T cell number (Fig. 4d, e).

In addition to these quantitative changes in T cell numbers, there was a significant increase in activation marker expression, including PD1, on tumor-infiltrating CD8+ T cells (Fig. 4f). There were no significant treatment-mediated effects on tumor infiltrating CD4+ T cells or T cells isolated from the spleen (Additional file 2: Figure S4B and data not shown). Importantly, no significant differences between mmHERA-GITRL and mmHERA-GITRL (Fc+) treated mice were observed in anti-tumor efficacy or immune cell quantity or quality.

The single-agent anti-tumor activity of mmHERA-GITRL was also tested in the MC38-CEA model. MC38-CEA (C57Bl/6), like CT26wt (BALB/c), is a common colon carcinoma model, however syngeneic tumor studies are performed in a different mouse strain. The two distinct genetic backgrounds gave an opportunity to address tumor efficacy in the context of their propensities towards different immune polarization, for example, type 1 versus type 2 immunity. MC38-CEA tumor cells were implanted (s.c.) and mice were randomized into groups 5 days later with a mean primary tumor volume of approximately 65 mm^3^. All mice were treated (i.v.) with mmHERA-GITRL (1 mg/kg b.w.) or vehicle control (PBS) on days 5, 8, 12, 15 and 19. Treatment with mmHERA-GITRL resulted in 42% TGI compared to the control group (Additional file 2: Figure S5A, B).

### HERA-GITRL demonstrates significantly enhanced activity compared to a clinical benchmark agonistic anti-GITR antibody

Due to the role that many TNFRSF receptors play in enhancing anti-tumor immunity, multiple approaches have been developed to create TNFRSF receptor agonists. The most common approach has been to generate anti-receptor antibodies. Although antibodies have been shown to be effective at blocking receptor-ligand interactions, they have mixed results as TNFRSF receptor agonists due to having only two target-binding sites (i.e., GITR-binding sites) and an inability to recreate the 3D organization of the natural trimeric ligand/receptor assembly complex.

**Fig. 5:**
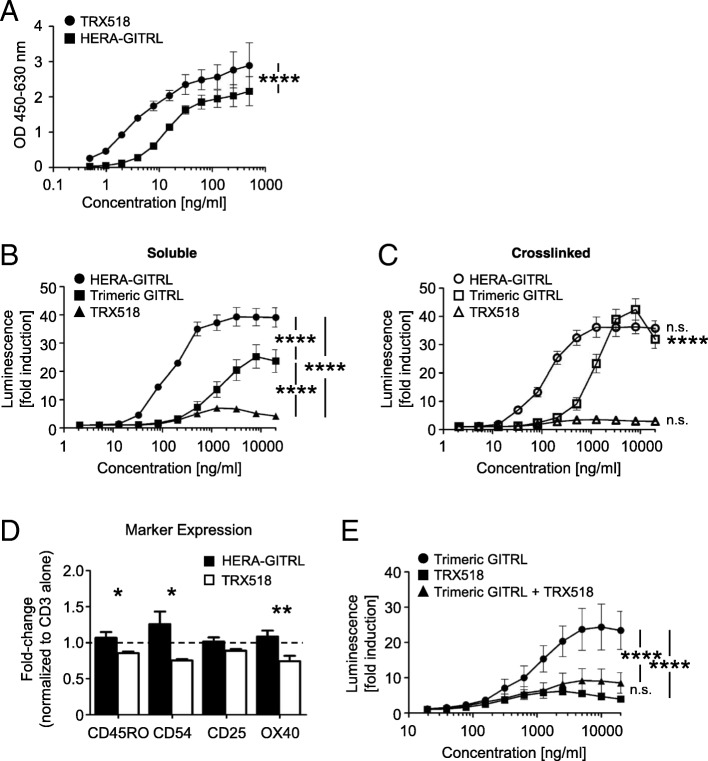
HERA-GITRL demonstrates significantly enhanced agonistic activity compared to a clinical benchmark antibody. **a** ELISA showing binding of hexavalent HERA-GITRL and bivalent anti-GITR antibody (TRX518) to immobilized human GITR-Fc. TRX518 bound to human GITR-Fc was detected by goat anti-human IgG-Peroxidase. Values are mean OD (*n* = 3) ± S.D. Representative data from at least three independent experiments are shown. The p value represents a comparison between samples using a two-way ANOVA. ****p < 0.0001. **b, c** NFκB-luc2/GITR-expressing Jurkat cells were incubated with the indicated concentrations of HERA-GITRL, trimeric GITRL or anti-GITR antibody, without (**b**) or with (**c**) crosslinking, at 37 °C for six hours. Data are shown as mean values (± S.D.) from three independent experiments. **b** The p values represent a comparison between samples using a two-way ANOVA. ****p < 0.0001. **c** The p values represent a comparison between soluble (**b**) and crosslinked (**c**) samples using a two-way ANOVA. ****p < 0.0001. n.s. not significant. **d** Naïve CD4+ T cells  were isolated from the peripheral blood of healthy volunteers and stimulated with anti-CD3 antibody (1 μg/mL) in the presence of HERA-GITRL or anti-GITR antibody (both 1000 ng/mL). On day five, T cells were harvested and examined by FCM. Due to high donor variability observed when comparing PBS to anti-CD3 antibody treatment, all values have been normalized to the anti-CD3 antibody alone group. The dashed line indicates the 1.0 value. The p values represent a comparison between samples using a one-way ANOVA. *p < 0.05, **p < 0.01. **e** NFκB-luc2/GITR-expressing Jurkat cells were incubated with the indicated concentrations of trimeric GITRL and/or anti-GITR antibody at 37 °C for six hours. A constant ratio by mass (1:1) was maintained in the combination group. Data are shown as mean values (± S.D.) from three independent experiments. The p values represent a comparison between samples using a two-way ANOVA. ****p < 0.0001. n.s. not significant

As we have shown in Fig. 1e, the hexavalent HERA-GITRL induces significantly higher receptor signaling activity compared to the trivalent GITRL. To test the hypothesis that a bivalent antibody would have even more trouble clustering a sufficient number of receptors to transmit a productive signal, the clinical benchmark anti-human GITR antibody (TRX518) was purchased to perform in vitro comparison studies. HERA-GITRL and the anti-human GITR antibody (TRX518) were tested for binding to recombinant human GITR-Fc by ELISA (Fig. 5a). The anti-GITR antibody demonstrated significantly stronger binding than HERA-GITRL, which is consistent with the known binding characteristics of antibodies and TNFSF ligands.

In order to compare the activity of the hexavalent HERA-GITRL and trivalent GITRL to the bivalent antibody, we tested productive GITR signaling using a reporter cell assay. NFκB-luc2/GITR-expressing Jurkat cells were incubated with various concentrations of HERA-GITRL, GITRL or anti-GITR antibody and after six hours of culture, luminescence was measured. As shown in Fig. 5b, treatment with HERA-GITRL resulted in significantly higher reporter activity compared to the anti-GITR antibody across a wide range of concentrations. Even the trimeric GITRL showed significantly higher activity than the benchmark antibody. Since it has been demonstrated that the biological activity of many therapeutic antibodies relies on secondary crosslinking via FcγR interactions, the GITR reporter cell assay was also performed with crosslinked HERA-GITRL, GITRL and anti-GITR antibody. In this assay, multiple “secondary” anti-human Ig antibodies were used to crosslink the benchmark anti-GITR antibody. In all cases, crosslinking did not increase the activity of the anti-GITR antibody (Fig. 5c and data not shown). Crosslinking also failed to increase the activity of HERA-GITRL. The hexavalent layout of HERA-GITRL appears to result in optimal biological activity of this molecule while a potential risk for detrimental hyper-activation of GITR seems to be negligible. In contrast, crosslinking significantly increased the activity of the trimeric GITRL.

In order to compare biological activity of the hexavalent HERA-GITRL to the bivalent anti-GITR antibody, naïve CD4+ T cells were stimulated with plate-bound anti-CD3 antibody (1 μg/mL) in the presence of HERA-GITRL or anti-GITR antibody (both 1000 ng/mL). On day five, T cells were examined for evidence of activation and differentiation. Treatment with HERA-GITRL resulted in a significantly higher percentage of T cells expressing CD45RO, CD54 and OX40 compared to treatment with the anti-GITR antibody (Fig. 5d). Interestingly, while HERA-GITRL consistently increased T cell activity compared to anti-CD3 antibody alone, the anti-GITR antibody consistently reduced the T cell response. In order to understand how treatment with a supposedly agonistic clinical benchmark antibody could result in decreased T cell activation, the GITR reporter assay was used to examine signaling when the natural ligand (represented by the trimeric GITRL) was co-incubated with the anti-GITR antibody. GITR-reporter cells were incubated with various concentrations of trimeric GITRL plus anti-GITR antibody (maintaining a constant mass ratio). As shown in Fig. 5e, the presence of the anti-GITR antibody significantly inhibits the activity of the trimeric GITRL across a wide range of concentrations, indicating a blockade of the GITR-ligand binding site by the antibody.

## Discussion

GITR has been a key immunotherapeutic target for almost 20 years and multiple strategies to induce GITR signaling have been investigated. The limited clinical efficacy of GITR agonists results from structural and functional characteristics of antibodies, including the presence of only two target-binding domains per molecule, that are unsuitable for stimulating the TNFRSF. Here we describe the engineering and production of a novel hexavalent agonist of human GITR. The lab-scale HERA-GITRL production process yielded defined and stable products devoid of contaminating proteins and product related aggregates. In addition, the stability of HERA-GITRL was tested under a variety of different conditions and found to be suitable for standard large-scale production processes. Binding studies confirmed previously published reports that the purified human HERA-GITRL binds to human, and cynomolgus monkey, GITR but not mouse GITR [5]. These species-specific results confirmed that cynomolgus monkey is a relevant species for further studies and indicated the need for a specific mouse surrogate (mmHERA-GITRL).

To examine the biological activity of human HERA-GITRL, we isolated naïve CD4+ T cells and stimulated them in vitro. Consistently, treatment with the hexavalent HERA-GITRL significantly boosted proliferation and differentiation of stimulated T cells. Critically, these studies also demonstrated that treatment with HERA-GITRL alone did not produce any response in unstimulated T cells, suggesting that an antigen-specific and TCR-mediated signal is required for the GITR-mediated enhancement of T cell activity. This is a clear advantage over non-specific strategies, such as checkpoint inhibition or cell depletion-based therapies, that lead to serious immune-related adverse events (irAE) due to their broad mechanisms of action [19–21]. Because Treg cells play an important role in maintaining immune homeostasis and they express high levels of GITR we decided to investigate the effects of HERA-GITRL treatment on Treg cell activity. Previously published data have shown that, depending on the specific context, GITR signaling can have both inhibitory and stimulatory effects on Treg cell activity [22]. For example, “short-term” exposure to GITR stimulation leads to inhibition of Treg cell activity while “long-term” exposure stimulates their proliferation [22]. Treatment of Treg cells with HERA-GITRL did not influence Treg cell survival or proliferation. However, HERA-GITRL inhibited Treg cell-mediated suppression of effector T cells.

In order to assess pharmacokinetic and safety properties of HERA-GITRL, small-scale studies were conducted in healthy cynomolgus monkeys and mice (mmHERA-GITRL). These studies showed that HERA-GITRL had a terminal serum half-life of 1.5 days in cynomolgus monkeys and 7 h in mice (mmHERA-GITRL). Critically, no toxicological signs related to the administration of HERA-GITRL were observed during the in-life phase of these studies.

With respect to the intended usage as an immune-stimulatory agonist, the short half-life of HERA-GITRL can be considered an advantage over therapeutic antibodies. Unlike inhibitory or blocking therapies that require continuous prevention of specific receptor/ligand interactions, stimulatory strategies only need a short pulse for optimal activity. This is especially important for GITR stimulation where sustained/chronic GITR signaling preferentially activates regulatory T cells over effector T cells [22]. Antibodies, including the anti-GITR benchmark TRX518, have two to three week long half-lives. In order to avoid chronic stimulation or overstimulation, the exposure required for efficacy will need to be experimentally determined. The short half-life of HERA-GITRL makes it the ideal candidate for exploring this area.

The activity and safety of the hexavalent mmHERA-GITRL was also demonstrated in vivo using an antigen-specific adoptive transfer model. The hexavalent mmHERA-GITRL significantly boosted clonal expansion and differentiation of antigen-specific CD4+ and CD8+ T cells. Due to the potential for activation of non-specific and/or bystander T cells (such as, self-antigen-specific T cells), OVA-specific and non-specific T cells were examined. Even in the shared environment, where over 10% of CD4+ and CD8+ T cells were vigorously responding to their specific antigen, the non-specific (endogenous) T cells did not show any signs of activation. This system, which mimics a more realistic T cell response compared to in vitro stimulation with anti-CD3 antibodies, further demonstrated the specificity of HERA-GITRL. Having a requirement for an antigen-specific signal as well as having a clearly defined agonistic forward-signaling mechanism of action means that HERA-GITRL could have a superior safety profile compared to other approaches.

The single-agent activity of the hexavalent mmHERA-GITRL was demonstrated with the syngeneic mouse tumor models CT26wt and MC38-CEA. While the previous experiments showed antigen-specific T cell activation, it was important to demonstrate efficacy in tumor models. Treatment with mmHERA-GITRL resulted in dose-dependent tumor-growth inhibition and significant changes in the tumor-infiltrating T cell compartment. Specifically, there was an increase in the number and activation status of T cells found in the tumor tissue. HERA-GITRL has been designed to be a true agonist and, therefore, effective in the absence of FcγR-binding functionality. The CT26wt  tumor model was also used to determine if FcγR binding, a common prerequisite for “agonistic” antibody activity, could increase the anti-tumor effects of HERA-GITRL. Importantly, no significant differences between mmHERA-GITRL and mmHERA-GITRL (Fc+) treated mice were observed in anti-tumor efficacy or immune cell activity underlining that efficacy of mmHERA-GITRL is independent from FcγR-binding functionality.

Due to the role that TNFRSF receptors play in enhancing anti-tumor immunity, multiple approaches have been developed to create TNFRSF receptor agonists. While antibodies are effective at blocking receptor-ligand interactions and stimulating the dimeric receptors of the immunoglobulin (B7/CD28) superfamily, they have mixed results as TNFRSF receptor agonists [9]. The limited clinical efficacy of GITR agonists results from structural and functional characteristics of antibodies, including the presence of only two target (GITR)-binding sites per molecule, that are unsuitable for stimulating the TNFRSF. To test the hypothesis that an antibody would have even more trouble clustering a sufficient number of receptors to transmit a productive signal, the activity of the hexavalent HERA-GITRL, a trivalent GITRL and a bivalent clinical benchmark anti-human GITR antibody was compared. In a GITR-signaling-based reporter assay as well as a primary T cell activation assay, treatment with HERA-GITRL resulted in significantly higher activity compared to the anti-GITR antibody. Even the trimeric GITRL showed significantly higher signaling activity than the benchmark antibody. Since secondary crosslinking has been shown to influence the activity of agonistic antibodies [10, 23, 24], the GITR signaling assay was also performed in the presence of crosslinking. Predictably, the crosslinked trimeric GITRL showed higher signaling activity. In contrast, crosslinking did not increase the activity of the anti-GITR antibody.

These results support the concept that bivalent compounds, like antibodies, do not have the correct structural and functional characteristics to generate a productive signal [10, 13, 14, 19]. The data also support our previous published findings with HERA-TRAIL, HERA-CD40L and HERA-CD27L suggesting that proper TNFRSF receptor clustering, best achieved by higher order structures based on the natural ligand, is required for full induction of down-stream signaling and function [13–15]. It is important to mention that while these conclusions are true for human TNFRSF signaling, comparisons between trivalent ligand-based concepts and bivalent antibodies are more complicated in mice. Specifically, crystal structure data has shown that murine GITRL can exist and function as a dimer, highlighting important differences between human and mouse [12, 25, 26]. Since murine GITR can signal as a trimer or a dimer, a bivalent antibody should be able function as a GITR agonist. As an additional complication, the benchmark anti-mouse GITR antibody (clone DTA-1) is a rat IgG2b that causes severe anaphylaxis when repeatedly injected into mice [27]. Unfortunately, even “murinized” DTA-1 antibodies can cause anaphylaxis due to mouse strain differences in IgG2 expression [28].

Interestingly, while HERA-GITRL consistently increased T cell activity compared to anti-CD3 antibody alone, the clinical benchmark anti-GITR antibody consistently reduced the T cell response. In order to understand how treatment with an “agonistic” antibody could result in decreased T cell activation, the GITR reporter assay was used to examine signaling when the natural ligand (represented by the trimeric GITRL) was co-incubated with the anti-GITR antibody. The presence of the anti-GITR antibody significantly inhibited the activity of the trimeric GITRL, which provided further evidence that antibodies can interfere with the anti-tumor immune response by blocking the natural TNFSF/TNFRSF signaling pathways [14]. In fact, it has been demonstrated that this clinical benchmark antibody blocks binding of the natural ligand to GITR [22].

In addition to blocking the activity of natural ligand, the structural and functional characteristics of antibodies can lead to a series of unnecessary complications. For example, the majority of antibodies targeting the TNFRSF are fully dependent on additional crosslinking, generally via the Fc domain of the antibodies, for activity [10, 23, 24]. While these interactions provide some activity, there are serious side effects associated with the functional FcγR-binding domain. This includes depletion of target-expressing cells via ADCC and ADCP as well as antibody-dependent cytokine release (ADCR) and cytokine release syndrome (CRS) [19, 24, 29–31]. In fact, Fc/FcγR interactions have been shown to be responsible for many of the irAEs associated with antibody-based immunotherapy.

## Conclusions

For many years, various strategies have been employed targeting GITR signaling for cancer therapy. In fact, the first GITR-targeting clinical trial began almost 10 years ago. The limited clinical efficacy of GITR agonists is primarily due to the structural and functional characteristics of antibodies, including the presence of only two target-binding domains per molecule, that are unsuitable for stimulating the TNFRSF. Here, we describe the development of HERA-GITRL, a true GITR agonist with a clear forward-signaling mechanism of action. HERA-GITRL shows single agent anti-tumor activity both in vitro and in vivo. The hexavalent HERA-GITRL has superior activity compared to a bivalent clinical benchmark antibody. Importantly, this agonistic activity is not dependent on FcγR-based mechanisms.

Cancer immunotherapy is currently being dominated by immune checkpoint inhibitors and over 1500 clinical trials are registered targeting PD-1, PD-L1 or CTLA-4. The effectiveness of these approaches is dependent on the presence of previously primed anti-tumor T cells. Immune-stimulatory compounds, such as HERA-GITRL, are able to boost T cell priming and, therefore, generate effective anti-tumor immunity that should also work well in combination with immune checkpoint inhibitors.

## Additional files

Additional file 1: Supplementary Methods. (DOCX 45 kb)

Additional file 2: **Figure S1** Purification and biochemical features of mmHERA-GITRL. **Figure S2** Binding and pharmacokinetic comparison of mmHERA-GITRL and mmHERA-GITRL (Fc+). **Figure S3** Pharmacokinetics of HERA-GITRL in mouse and cynomolgus monkey. **Figure S4** mmHERA-GITRL demonstrates single-agent in vivo efficacy that is independent of Fcγ-receptor binding. **Figure S5** mmHERA-GITRL demonstrates significant in vivo efficacy in mice as a single agent in the MC38-CEA tumor model. **Table S1** Summary of HERA-GITRL stability under various conditions. **Table S2** Summary of the binding constants of HERA-GITRL to human, mouse and cynomolgus monkey GITR-Fc. **Table S3** Summary of the pharmacokinetic properties of HERA-GITRL in mouse and cynomolgus monkey. **Table S4** Summary of the pharmacokinetic properties of mmHERA-GITRL and mmHERA-GITRL (Fc+) in mouse. (PDF 866 kb)

## Data Availability

Data are available in the main text and supplementary materials, raw data available upon request.

## Abbreviations

ADCC: Antibody-dependent cell-mediated cytotoxicity
ADCP: Antibody-dependent cell-mediated phagocytosis
ADCR: Antibody-dependent cytokine release
AFC: Affinity purification
APC: Antigen-presenting cell
ATRA: All-trans retinoic acid
b.w.: Body weight
CFSE: Carboxyfluorescein succinimidyl ester
CHO-S: Chinese Hamster Ovary cell, suspension liquid culture
CRS: Cytokine release syndrome
CTV: Cell trace violet
FCM: Flow cytometry
FcγR: Fcγ receptor
HERA: Hexavalent TNF receptor agonist
HMWS: High molecular weight species
i.p.: Intraperitoneal
i.v.: Intravenous
irAE: Immune-related adverse event
K_D_: Equilibrium binding constant
LMWS: Low molecular weight species
LN: Lymph node
mmHERA-GITRL: Mouse surrogate HERA-GITRL
OVA: Chicken ovalbumin
PBMC: Peripheral blood mononuclear cells
PK: Pharmacokinetic
RBD: Receptor-binding domain
s.c.: Subcutaneous
sc: Single chain
scGITRL-RBD: Single-chain GITRL-receptor-binding-domain
SEC: Size-exclusion chromatography
TCR: T cell receptor
TGI: Tumor-growth inhibition
TNFRSF: Tumor necrosis factor receptor superfamily
TNFRSF18, GITR, CD357: Glucocorticoid-induced TNFR-related protein
TNFSF: Tumor necrosis factor superfamily
TNFSF18, GITRL: GITR ligand
Treg: Regulatory T cell
