# Supplementary material for: HERA-GITRL activates T cells and promotes anti-tumor efficacy independent of FcγR-binding functionality
Source: J Immunother Cancer. 2019 Jul 19;7:191. doi: 10.1186/s40425-019-0671-4 (PMC6642547; doi:10.1186/s40425-019-0671-4)
Supplement: Supplementary file 1 — Supplementary Methods. (DOCX 45 kb) [file 40425_2019_671_MOESM1_ESM.docx]

# Additional file 1: Supplementary Methods

Journal for ImmunoTherapy of Cancer - Research article

**Title**. HERA-GITRL activates T cells and promotes anti-tumor efficacy independent of FcγR-binding functionality

**Authors and affiliations**

David M. Richards, Viola Marschall^1^, Katharina Billian-Frey, Karl Heinonen, Christian Merz, Mauricio Redondo Müller, Julian P. Sefrin, Matthias Schröder, Jaromir Sykora, Harald Fricke^2^, Oliver Hill, Christian Gieffers and Meinolf Thiemann*

Research and Development, Apogenix AG, Im Neuenheimer Feld 584, 69120 Heidelberg, Germany

^1^ Current address: Biotest AG, Dreieich, Germany

^2^ Current address: SOTIO, Prague, Czech Republic

*Corresponding author

Dr. Meinolf Thiemann, Director Protein Analytics

Email: meinolf.thiemann@apogenix.com

Running title: Hexavalent GITRL promotes anti-tumor immunity

# Supplementary Methods

## Construction, expression and purification of hexavalent HERA-GITRL

To engineer a hexavalent GITR agonist, we designed a single-polypeptide chain with three copies of a GITRL protomer sub-sequence. Specifically, three copies from human GITRL comprising amino acids E56-S177 were interconnected with two glycine-serine based linkers 8 amino acids in length. The resulting trivalent single chain-GITRL-receptor-binding-domain (scGITRL-RBD) was fused to the Fc-part of a human IgG1-mutein, which is deficient for Fcγ receptor binding, to create an Fc-silenced hexavalent scGITRL-RBD dimer. In addition, HERA-GITRL engineering prototype contains a C-terminal Strep-Tag II for laboratory purification purposes. Secretory pathway-based expression was achieved by adding an appropriate signal-peptide to the N-terminus. Codon optimized synthetic genes were obtained from GeneWiz, re-cloned into proprietary expression vectors and transfected into suspension-adapted Chinese Hamster Ovary cells (CHO-S cells, Invitrogen).

HERA-GITRL content of the supernatants was monitored by ELISA during the selection process and the best-expressing cell pools were subsequently expanded for protein production. For expression and purification, high titer cell pools were expanded in a WAVE bioreactor (GE Healthcare) at 37°C with 7% CO_2_ for seven to twelve days in chemically defined medium (PowerCHO2-CD, Lonza) with two feeds (PowerFeed A, Lonza). Cells were harvested when cell viability dropped below 70% and the supernatant was clarified by centrifugation and filtration prior to purification. The hexavalent HERA-GITRL constructs were purified by a two-step process combining Streptactin affinity purification (AFC) followed by preparative size-exclusion chromatography (SEC) both performed under physiological buffer conditions at pH 7.4. Purity and integrity of the protein was confirmed by analytical SEC as well as denaturing and non-denaturing SDS-PAGE.

## Construction, expression and purification of HERA-GITRL mouse surrogates (mmHERA-GITRL)

To engineer HERA-GITRL mouse surrogates (mmHERA-GITRL), three copies from mouse GITRL comprising amino acids S43-S177 were used to generate a mouse scGITRL-RBD that was fused to the Fc-domain of a mouse IgG2A-mutein, deficient for Fcγ receptor binding, to create an Fc-silenced hexavalent scGITRL-RBD dimer. A second mouse surrogate, with functional Fcγ receptor binding (labeled mmHERA-GITRL Fc+) was also generated with the wildtype IgG2A Fc domain. Mouse surrogates were cloned, expressed and purified as described above for HERA-GITRL.

## GITR signaling and biological activity reporter assay

We evaluated GITR signaling *in vitro* following treatment with hexavalent HERA-GITRL, trimeric GITRL or a clinical benchmark anti-human GITR antibody by measuring luciferase activity in a GITR-specific cell-based bioassay (NFκB-luc2/GITR Jurkat cell bioassay, Promega GmbH). The trimeric GITRL was purchased as “Recombinant Human GITR Ligand/TNFSF18 Protein” from R&D Systems, Inc. (Minneapolis, MN, USA). The anti-TNFRSF18 Therapeutic Antibody (TRX518) was purchased from Creative Biolabs (Shirley, NY, USA). NFκB-luc2/GITR-expressing Jurkat cells were plated in a 96-well plate and incubated briefly at 37°C prior to addition of the indicated concentrations of HERA-GITRL, trimeric GITRL or anti-GITR antibody. For cross-linking experiments, equal amounts of the following antibodies were added: HERA-GITRL was cross-linked with StrepMab Immo (IBA, cat. no. 2-15-17-001), trimeric GITRL was cross-linked with anti-His Tag antibody (BioLegend, cat. no. 652502) and TRX518 was cross-linked with anti-human Fc antibody (Dianova, cat. no. 209-005-098). Productive GITR signaling induced by treatment with the agonistic compounds drives expression of firefly luciferase in the NFκB-luc2/GITR Jurkat cells. After six hours of induction at 37°C, the luciferase assay reagent was added and luminescence (RLU) was measured (Tecan Infinite F500). The fold induction of measured luminescence was calculated by the formula: RLUstimulated / RLUunstimulated control in order to compare multiple experiments.

## Functional binding of hexavalent murine and human HERA-GITRL to human, mouse and cynomolgus monkey GITR-Fc and binding of TRX518 to human GITR-Fc

For ELISA assessing functional binding of HERA-GITRL, the mouse surrogate mmHERA-GITRL or the anti-GITR antibody (TRX518) to its corresponding receptor GITR, microtiter plates were coated with 1 µg/mL human or 2 µg/mL mouse GITR-Fc (Bio-Techne GmbH) or 2 µg/mL cynomolgus monkey GITR-Fc. Cynomolgus monkey (*Macaca fascicularis*) GITR-Fc fusion protein was generated in house and purified from CHO-S cells using the two-step protocol described above. After blocking plates with StartingBlock (Life Technologies), wells were incubated with the indicated concentrations of GITR agonists. Human HERA-GITRL bound to its corresponding receptor was detected via its Strep-Tag II employing Streptactin-Peroxidase (1:1000; IBA GmbH, Cat. No. 2-1502-001). Murine HERA-GITRL bound to its corresponding receptor was detected by goat anti mouse IgG-Peroxidase (1:1000; Sigma, Cat. No. 8924). The anti-GITR antibody TRX518 bound to human GITR-Fc was detected by goat anti human IgG-Peroxidase (1:1000; Jackson ImmunoResearch, Cat. No. 109-035-097). Subsequently, visualization of the bound GITR agonist was achieved via the converted Peroxidase-substrate TMB one (Kem-En-Tec Diagnostics) at a wavelength of 450 nm (with a 630 nm correction factor) in an ELISA reader (Thermo Mulitskan Ascent).

## In vitro T cell activation, proliferation and differentiation assays

To test the activity of HERA-GITRL on primary human T cells, naïve CD4+ T cells were isolated from peripheral blood mononuclear cells (PBMC) using indirect magnetic bead-based isolation kits (Cat. No. 130-094-131, Miltenyi). Purified T cells were labeled with carboxyfluorescein succinimidyl ester (CFSE, CFSE Cell Division Tracker Kit, BioLegend), resuspended in medium (AIM-V w/o FCS + AlbuMax, Gibco) and stimulated with pre-coated anti-CD3 antibody (overnight, clone OKT3, BioLegend, 1 µg/mL) or medium control. HERA-GITRL (10, 100 or 1000 ng/mL) was added immediately. On day five, T cells were harvested and examined by flow cytometry (FCM). For intracellular staining, cells were treated with PMA (20 ng/ml), Ionomycin (1 µM) and Brefeldin A (1:1000) at 37°C for 5 hours prior to being fixed, permeabilized, stained and examined by FCM.

## In vitro regulatory T cell activity assays

To test the activity of HERA-GITRL on human regulatory T cell survival, proliferation and suppressive function, natural regulatory T cells (Treg, CD4+ CD25+) were isolated from PBMC using a two-step magnetic bead-based isolation kit (Cat. No. 130-091-301, Miltenyi). For the Treg cell survival/proliferation assay, purified Treg cells were labeled with cell trace violet (CTV, Cat. No. C34557, ThermoFisher), resuspended in medium (X-VIVO, Cat. No. 04-418, Lonza) and stimulated with pre-coated anti-CD3 antibody (overnight, clone UCHT1, BioLegend, 0.5 µg/mL), soluble anti-CD28 antibody (clone CD28.2, BioLegend, 1 µg/mL), IL-2 (10 ng/mL) and all-trans retinoic acid (ATRA) (0.1 µM) or medium control. HERA-GITRL (10, 100 or 1000 ng/mL) or vehicle control (PBS) was added immediately. On day five, T cells were harvested and examined by FCM. For the Treg cell-mediated suppression assay, Treg cells and responder T cells (Teff, CD3+ CD25-) were isolated by magnetic selection from the same donors. In this assay, the Teff cells were labeled with CTV. Treg cells were added to Teff cells at different ratios (Treg:Teff) and cells were stimulated and treated as described above. On day five, T cells were harvested and examined by FCM.

## Flow cytometry (FCM)

For flow cytometry (FCM), cells were labeled with the following antibodies (clone): anti-mouse CD3 (145-2C11); CD4 (RM4-5), CD8a (53-6.7), CD45.2 (104), CD44 (IM7) and CD279 (PD-1) (29F.1A12) and anti-human CD3 (UCHT1), CD4 (OKT4), CD8 (RPA-T8), CD25 (BC96), CD45RA (HI100), CD45RO (UCHL1), IFNγ (B27) and TNFα (MAb11) (all BD Bioscience or BioLegend). FoxP3/Transcription Factor staining buffer Set (Cat. No. 11500597, ThermoFisher). Cells were acquired using the FACSCelesta BVR12 (BD Biosciences) or Guava EasyCyte 12 Flow Cytometer (EMD Millipore). Antibody quality was checked and gating was performed using isotype controls. FlowJo Software (version 10) (FlowJo, LLC) was used for the analysis of FCM data.

## Storage, freeze/thaw, heat stress and pH stability assays

For storage stability, HERA-GITRL was stored at 37°C ± 2°C (one day, five days, two weeks), room temperature (two weeks) or 5°C ± 3°C (two weeks) before stability analysis. For freeze/thaw stability, HERA-GITRL was frozen at <-15°C and subsequently thawed at room temperature. Samples were exposed to one, three or five additional freeze/thaw cycles before stability analysis. For pH stability, HERA-GITRL was exposed to pH 3.0, pH 3.4, pH 3.6, pH 3.8 or pH 4.0 (300 mM Na-citrate/HCl) (Sörensen), pH 7.0 (300 mM phosphate) (Sörensen) or pH 10.0, pH 11.0, pH 12.0 (300 mM glycine/NaOH, 300 mM NaCl) (Sörensen). At 2 hours or 4 hours after re-buffering, aliquots were taken and frozen at <- 65°C prior to stability analysis. For heat stress, HERA-GITRL was exposed for 10 minutes in a thermo-block to the following temperatures: 50°C, 54°C, 58°C, 62°C, 66°C. After exposure to heat and storing these samples at <-15°C, various analytics were performed employing non-heated HERA-GITRL as control.

Procedures used to assess the stability of HERA-GITRL included analytical SEC (HPLC), SDS-PAGE, thermal shift stability assay and determination of binding to the receptor GITR with an ELISA (described above). Analytical SEC of protein samples was performed employing the HPLC device from Agilent (1260 Infinity). Peak heights and peak areas for the main peak as well as for high molecular weight species (HMWS) and low molecular weight species (LMWS) peaks were determined and relative quantities were calculated. For thermal shift stability assays, protein samples were analyzed employing SyPro Orange as a fluorescent dye. In a PCR thermal cycler (MiniOpticon MJ, BioRad), samples were heated from 25°C to 95°C with 1°C increments. Fluorescence was monitored at each temperature interval and the melting points of the samples were determined.

## Determination of pharmacokinetic parameters of HERA-GITRL and mmHERA-GITRL in mice and cynomolgus monkeys

Female CD1 mice or male cynomolgus monkeys were administered with 1 or 10 mg/kg body weight (b.w.) of HERA-GITRL or 1 mg/kg b.w. of the mouse surrogate (mmHERA-GITRL) as a single intravenous (i.v.) injection and whole blood was collected up to 336 hours after test item administration. Serum was prepared and HERA-GITRL or mmHERA-GITRL serum concentrations were quantitated by ELISA (assay principle as described above) assessing functional binding of HERA-GITRL to human GITR-Fc or mmHERA-GITRL to mouse GITR-Fc. ELISA assays were carried out using reference HERA-GITRL or mmHERA-GITRL as calibration and control samples. The measured data of the standard concentrations were used to create calibration curves using a five-parameter fit. This enabled the determination of the unknown HERA-GITRL or mmHERA-GITRL concentrations in the respective serum samples. Pharmacokinetic (PK) parameters were calculated using the program PK Solutions Version 2.0 for non-compartmental PK data analysis (Summit Research Services). All experimental protocols were approved by the Ethics Committee for Animal Experimentation.

## OT-I CD8+ and OT-II CD4+ T cell adoptive transfer – analysis of antigen-specific T cell activation in vivo

In order to measure antigen-specific T cell responses, we adoptively transferred 2 x 10^6^ T cells from each of the T cell receptor transgenic “OT-I” donor mice (CD8+) and “OT-II” donor mice (CD4+) into female Ly5.1 recipient mice (all C57Bl/6 background) [1-3]. These donor mice have T cells that specifically recognize individual peptides derived from chicken ovalbumin (OVA) in the context of MHC class I (OT-I) and MHC class II (OT-II). The recipient mice were challenged with OVA intraperitoneal (i.p.) together with i.v. treatment with the different compounds or vehicle control. Analysis of OT-I CD8+ and OT-II CD4+ T cell proliferation was done with whole blood and spleen samples from recipient mice that were injected with OT-I/OT-II cells on day -1 and challenged OVA (i.p.) on day 0 and treated with HERA-GITRL (1 or 5 and 1 or 8 mg/kg b.w.) or vehicle control (PBS) on day 0. For OT-I/OT-II T cell identification on days 6, 10 and 13, we used CD8/CD4 and a congenic marker CD45.2. All experimental protocols were approved by the Ethics Committee for Animal Experimentation.

## Analysis of the anti-tumor effect of mmHERA-GITRL in vivo (syngeneic mouse models)

The CT26wt anti-tumor efficacy experiment was performed using 6-week-old female BALB/c mice (Strain BALB/cAnNCrl, Charles River). Freshly cultured CT26wt tumor cells (5 x 10^5^ in 100 µl RPMI) were implanted (day 0) (subcutaneous, s.c.) into the right flank of all mice. Eleven days after tumor inoculation, mice were randomized into groups of six mice per treatment group with a mean primary tumor volume of 101 mm^3^ (range: 79 to 117 mm^3^). All mice were treated twice weekly (four administrations total on days 11, 14, 18 and 21) starting on the day of randomization. Vehicle control mice were treated i.v. with 10 mL/kg b.w. PBS. Both, mmHERA-GITRL and mmHERA-GITRL (Fc+) mice were treated i.v. at a dose of 1 or 10 mg/kg b.w. The in-life phase of the study finished on day 23 following tumor implantation.

The MC38-CEA anti-tumor efficacy experiment was performed using 6-week-old female C57Bl/6 mice (Strain C57Bl/6NAnNCrl, Charles River). Freshly cultured MC38-CEA tumor cells (1 x 10^6^ in 100 µl PBS) (day 0) were implanted s.c. into the right flank of all mice. Five days after tumor inoculation, mice were randomized into groups of six mice per treatment group with a mean primary tumor volume of approximately 65 mm^3^ (range: 30 to 120 mm^3^). All mice were treated twice weekly (five administrations total on days 5, 8, 12, 15 and 19) starting on the day of randomization. Vehicle control mice were treated i.v. with 10 mL/kg b.w. PBS. mmHERA-GITRL mice were treated i.v. at a dose of 1 mg/kg b.w. The in-life phase of the study finished on day 22 following tumor implantation. One animal in the control group was terminated early due to ethical considerations that was independent of tumor size (observed ulceration).

Tumor volume was determined twice weekly by caliper measurement. At the end of the studies, tumor samples and spleens were harvested and tumor weights were measured. All experimental protocols were approved by the Ethics Committee for Animal Experimentation.

## Tissue isolation and sample preparation from in vivo assays

For tissue isolation, spleens, tumors and lymph nodes (LN; axillary, brachial, and inguinal) were removed and organs were processed with a gentleMACS Dissociator (Miltenyi) and filtered to prepare single-cell suspensions. Whole venous blood was collected in lithium-heparin coated capillary tubes. Ammonium chloride–potassium bicarbonate lysis buffer was used to lyse erythrocytes in spleen and blood samples. AccuCheck+ Counting beads (Invitrogen) were added to tumor samples before acquisition, and cell counts were determined according to manufacturer’s directions.

## Determination of equilibrium binding constants (K_D_)

The equilibrium binding constant (K_D_) of HERA-GITRL with human, mouse and cynomolgus monkey GITR-Fc were calculated based on kinetic binding data (k_on_ and k_off_) determined with an automated biosensor system (Attana A100). For this purpose, the respective recombinant human, mouse and cynomolgus GITR-Fc were immobilized on the surface of a carboxyl-activated QCM-chip. HERA-GITRL was used as a soluble analyte at different concentrations. Binding and dissociation was analyzed in real time, and the respective K_D_ was calculated.

## Statistics

Statistical analysis of all experimental data was performed using Prism software (GraphPad). The statistical tests utilized are written in the figure legends. In general, one-way (or two-way for tumor growth studies) ANOVA tests were performed followed by post hoc Bonferroni multiple comparisons analysis. Results were considered statistically significant if p is less than 0.05.

# References

1. Barnden MJ, Allison J, Heath WR, Carbone FR. Defective Tcr Expression in Transgenic Mice Constructed Using Cdna-Based Alpha- and Beta-Chain Genes under the Control of Heterologous Regulatory Elements. Immunol Cell Biol. 1998;76(1):34-40.

2. Hogquist KA, Jameson SC, Heath WR, Howard JL, Bevan MJ, Carbone FR. T Cell Receptor Antagonist Peptides Induce Positive Selection. Cell. 1994;76(1):17-27.

3. Kearney ER, Pape KA, Loh DY, Jenkins MK. Visualization of Peptide-Specific T Cell Immunity and Peripheral Tolerance Induction in Vivo. Immunity. 1994;1(4):327-39.
