# Supplementary material for: HERA-GITRL activates T cells and promotes anti-tumor efficacy independent of FcγR-binding functionality
Source: J Immunother Cancer. 2019 Jul 19;7:191. doi: 10.1186/s40425-019-0671-4 (PMC6642547; doi:10.1186/s40425-019-0671-4)
Supplement: Supplementary file 2 — Figure S1 Purification and biochemical features of mmHERA-GITRL. Figure S2 Binding and pharmacokinetic comparison of mmHERA-GITRL and mmHERA-GITRL (Fc+). Figure S3 Pharmacokinetics of HERA-GITRL in mouse and cynomolgus monkey. Figure S4 mmHERA-GITRL demonstrates single-agent in vivo efficacy that is independent of Fcγ-receptor binding. Figure S5 mmHERA-GITRL demonstrates significant in vivo efficacy in mice as a single agent in the MC38-CEA tumor model. Table S1 Summary of HERA-GITRL stability under various conditions. Table S2 Summary of the binding constants of HERA-GITRL to human, mouse and cynomolgus monkey GITR-Fc. Table S3 Summary of the pharmacokinetic properties of HERA-GITRL in mouse and cynomolgus monkey. Table S4 Summary of the pharmacokinetic properties of mmHERA-GITRL and mmHERA-GITRL (Fc+) in mouse. (PDF 866 kb) [file 40425_2019_671_MOESM2_ESM.pdf]

Additional file 2: Supplementary Figures and Tables

Journal for ImmunoTherapy of Cancer - Research article

Title. HERA-GITRL activates T cells and promotes anti-tumor efficacy independent of FcγR-binding functionality

Authors and affiliations
David M. Richards, Viola Marschall[1], Katharina Billian-Frey, Karl Heinonen, Christian Merz, Mauricio Redondo Müller, Julian P. Sefrin, Matthias Schröder, Jaromir Sykora, Harald Fricke[2], Oliver Hill, Christian Gieffers and Meinolf Thiemann*

Research and Development, Apogenix AG, Im Neuenheimer Feld 584, 69120 Heidelberg, Germany
[1] Current address: Biotest AG, Dreieich, Germany
[2] Current address: SOTIO, Prague, Czech Republic

*Corresponding author
Dr. Meinolf Thiemann, Director Protein Analytics
Email: meinolf.thiemann@apogenix.com

Running title: Hexavalent GITRL promotes anti-tumor immunity

Figure S1. Purification and biochemical features of mmHERA-GITRL.
Figure S2. Binding and pharmacokinetic comparison of mmHERA-GITRL and mmHERA-GITRL (Fc+).
Figure S3. Pharmacokinetics of HERA-GITRL in mouse and cynomolgus monkey.
Figure S4. mmHERA-GITRL demonstrates single-agent *in vivo* efficacy that is independent of Fcγ-receptor binding.
Figure S5. mmHERA-GITRL demonstrates significant *in vivo* efficacy in mice as a single agent in the MC38-CEA tumor model.

Table S1. Summary of HERA-GITRL stability under various conditions.
Table S2. Summary of the binding constants of HERA-GITRL to human, mouse and cynomolgus monkey GITR-Fc.
Table S3. Summary of the pharmacokinetic properties of HERA-GITRL in mouse and cynomolgus monkey.
Table S4. Summary of the pharmacokinetic properties of mmHERA-GITRL and mmHERA-GITRL (Fc+) in mouse.

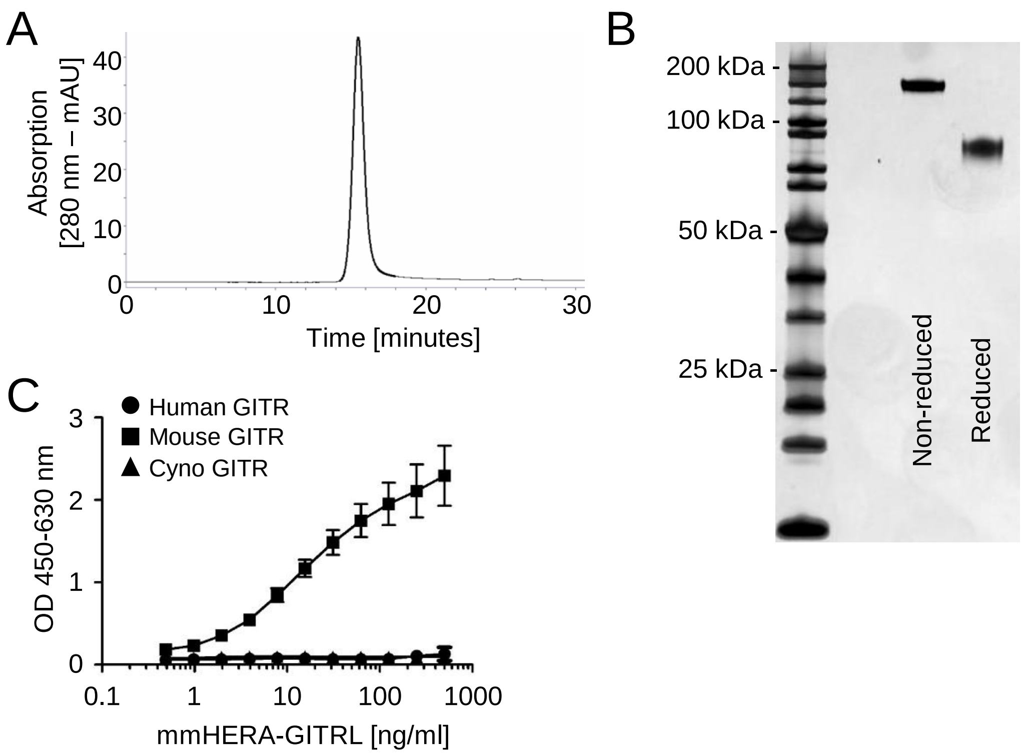

Figure S1. Purification and biochemical features of mmHERA-GITRL.

(a, b) Purification was accomplished by a two-step process combining AFC followed by preparative SEC. **(a)** For analytical SEC, purified mmHERA-GITRL was detected by online measurement of absorption at 280 nm. Content of monomer and aggregates was calculated as the AUC from the elution profile of the SEC. mmHERA-GITRL eluted as a single peak and showed no detectable aggregates.

(b) Purity and aggregation status of purified hexavalent mmHERA-GITRL was also assessed by non-reducing and reducing SDS-PAGE.

(c) ELISA showing binding of hexavalent mmHERA-GITRL to immobilized human, mouse and cynomolgus monkey GITR-Fc. Plate-bound GITR-Fc was probed with mmHERA-GITRL and the indicated concentrations. Receptor-bound mmHERA-GITRL was detected via a Strep-Tag II-specific antibody conjugated to horseradish peroxidase. Values are mean OD (n = 3) ± S.D. at a wavelength of 450 nm (with a 630 nm correction).

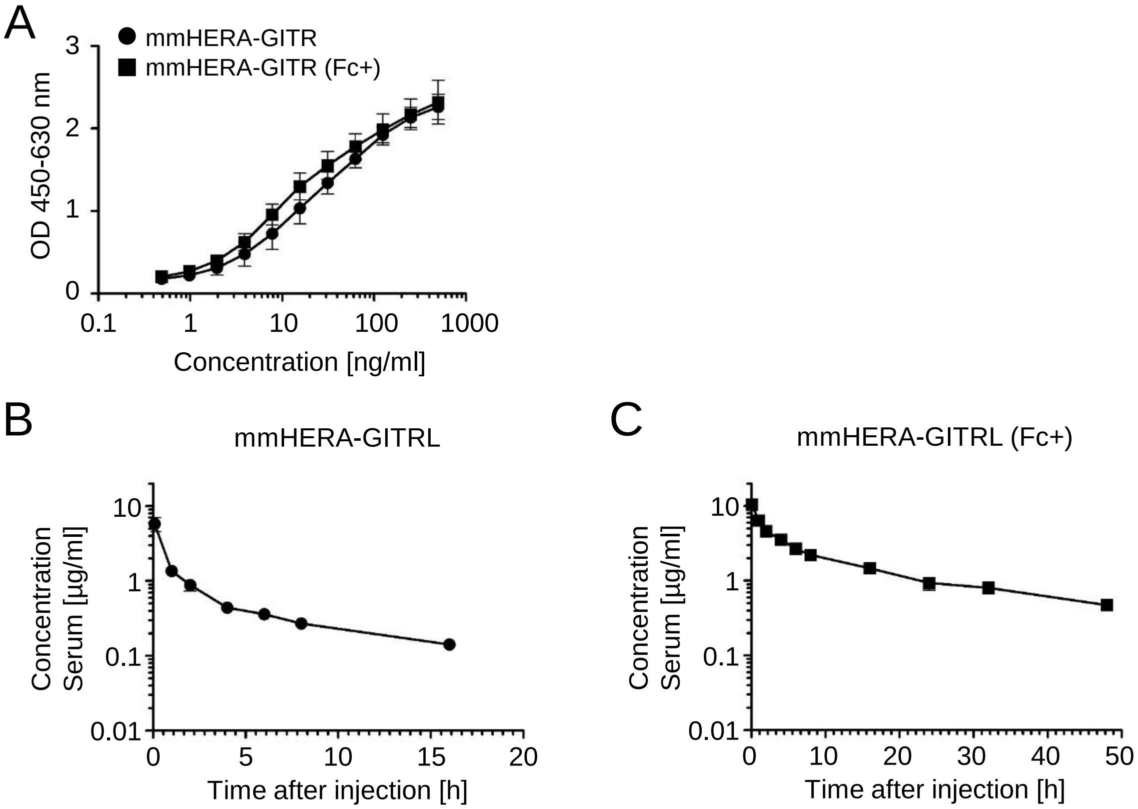

Figure S2. Binding and pharmacokinetic comparison of mmHERA-GITRL and mmHERA-GITRL (Fc+).

(a) ELISA showing binding of hexavalent mmHERA-GITRL and mmHERA-GITRL (Fc+) to immobilized mouse GITR-Fc. Plate-bound GITR-Fc was probed with the indicated formats and concentrations. Receptor-bound mmHERA-GITRL and mmHERA-GITRL (Fc+) were detected via a Strep-Tag II-specific antibody conjugated to horseradish peroxidase. Values are mean OD (n = 3) ± S.D. at a wavelength of 450 nm (with a 630 nm correction).

(b, c) Female CD1 mice were administered with 1 mg/kg b.w. of mmHERA-GITRL **(b)** or mmHERA-GITRL (Fc+) **(c)** as a single i.v. injection and whole blood was collected after test item administration. Serum was prepared and mmHERA-GITRL or mmHERA-GITRL (Fc+) serum concentration was quantitated by ELISA assays assessing functional binding of mmHERA-GITRL or mmHERA-GITRL (Fc+) to mouse GITR-Fc.

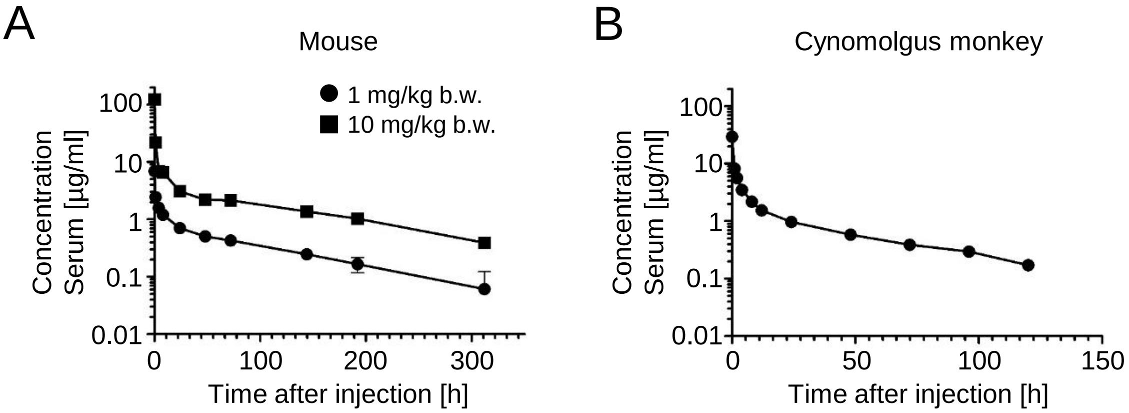

Figure S3. Pharmacokinetics of HERA-GITRL in mouse and cynomolgus monkey.

(a) Female CD1 mice were administered with 1 or 10 mg/kg b.w. of HERA-GITRL as a single i.v. injection and whole blood was collected after test item administration. Serum was prepared and HERA-GITRL serum concentration was quantitated by ELISA assays assessing functional binding of HERA-GITRL to human GITR-Fc.

(b) Male cynomolgus monkeys were administered with 1 mg/kg b.w. of HERA-GITRL as a single i.v. injection and whole blood was collected after test item administration. Serum was prepared and HERA-GITRL serum concentration was quantitated by ELISA assays assessing functional binding of HERA-GITRL to human GITR-Fc.

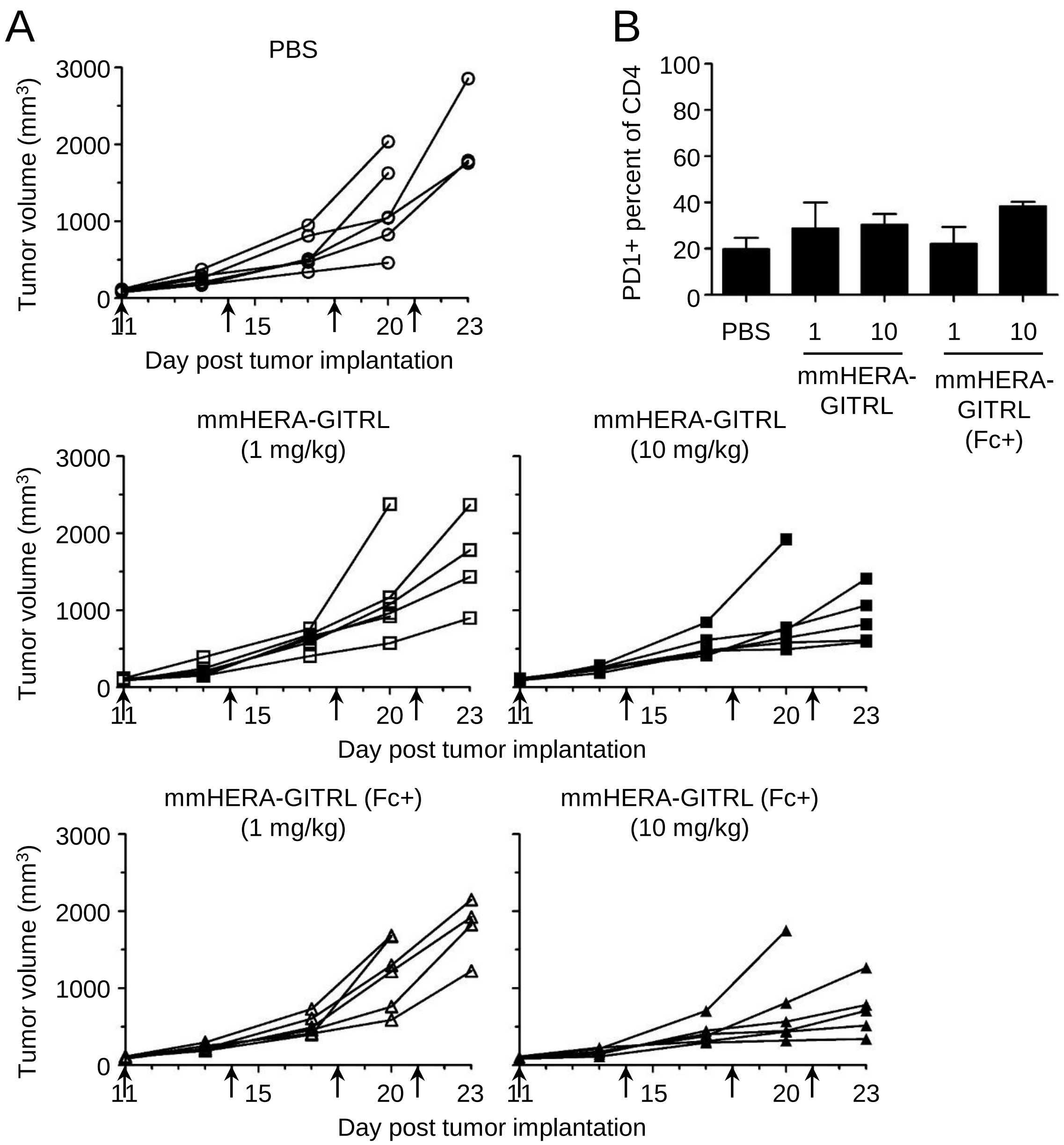

Figure S4. mmHERA-GITRL demonstrates single-agent *in vivo* efficacy that is independent of Fcγ-receptor binding.

(a, b) Freshly cultured CT26wt tumor cells (5 x 10^5 in 100 µl RPMI) were implanted s.c. into the right flank of 6-week-old female BALB/c mice. Tumor volume was determined twice weekly by caliper measurement. Mice were randomized on day 11 into groups of 6 mice per treatment group with a mean primary tumor volume of 101 mm^3. All animals were treated (i.v.) with mmHERA-GITRL, mmHERA-GITRL (Fc+) (both 1 or 10 mg/kg b.w.) or vehicle control (PBS) on days 11, 14, 18 and 21, indicated by the arrows. The in-life phase of the study finished on day 23 following tumor implantation. **(a)** Tumor size for all individual animals is shown. **(b)** On day 23, tumors were harvested, stained for surface marker expression and examined by flow cytometry. The percentage of PD1+ cells is shown for the CD4+ T cell population. A one-way ANOVA plus post hoc Bonferroni multiple comparisons test revealed no significant differences between groups.

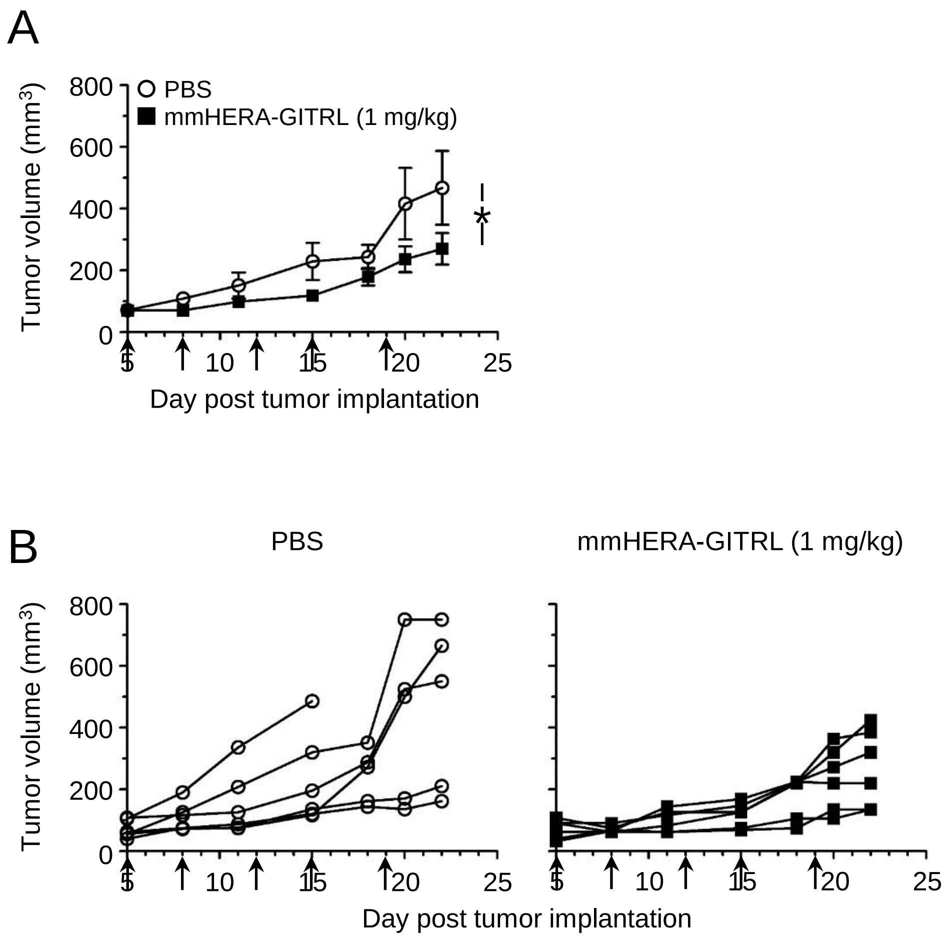

Figure S5. mmHERA-GITRL demonstrates significant *in vivo* efficacy in mice as a single agent in the MC38-CEA tumor model.

(a, b) Freshly cultured MC38-CEA tumor cells (1 x 10^6 in 100 µl PBS) were implanted s.c. into the right flank of 6-week-old female C57Bl/6 mice. Tumor volume was determined twice weekly by caliper measurement. Mice were randomized on day 5 into groups of 6 mice per treatment group with a mean primary tumor volume of 65 mm^3. All animals were treated (i.v.) with mmHERA-GITRL (1 mg/kg b.w.) or vehicle control (PBS) on days 5, 8, 12, 15 and 19, indicated by the arrows. The in-life phase of the study finished on day 22 following tumor implantation. **(a)** Each symbol represents the mean (n = 6) ± S.D. (**b**) The tumor size for all individual animals is shown. One animal in the control group was terminated early due to ethical considerations that was independent of tumor size (observed ulceration). A two-way ANOVA plus post hoc Bonferroni multiple comparisons analysis was conducted to compare the effects of treatment and time on tumor growth. *$p < 0.05$.

Table S1. Summary of HERA-GITRL stability under various conditions.

Stability	Condition	Results
Storage stability (up to 2 weeks)	5°C	2 weeks
	RT	2 weeks
	37°C	2 weeks
Freeze/thaw stability (up to 5 cycles)		5 cycles
Heat stress (10 min)		54 °C
pH stability	2 – 4 hours	pH 3.8 to pH 10

Table S2. Summary of the binding constants of HERA-GITRL to human, mouse and cynomolgus monkey GITR-Fc.

Receptor	Ligand	K_D
human GITR-Fc	HERA-GITRL	366 pM
mouse GITR-Fc	HERA-GITRL	(No binding)
cynomolgus monkey GITR-Fc	HERA-GITRL	602 pM

Table S3. Summary of the pharmacokinetic properties of HERA-GITRL in mouse and cynomolgus monkey.

Ligand	Species	Dosing [mg/kg b.w.]	$AUC_{0\text{-}inf}$ [µg · h/ml]	$t_{1/2}$ [h]
HERA-GITRL	CD-1 mice	1	113	86
HERA-GITRL	CD-1 mice	10	663	97
HERA-GITRL	Cynomolgus monkeys	1	123	37

Table S4. Summary of the pharmacokinetic properties of mmHERA-GITRL and mmHERA-GITRL (Fc+) in mouse.

Ligand	Species	Dosing [mg/kg b.w.]	$AUC_{0\text{-}inf}$ [µg · h/ml]	$t_{1/2}$ [h]
mmHERA-GITRL	CD-1 mice	1	11	7.4
mmHERA-GITRL (Fc+)	CD-1 mice	1	87	18.3
